# Social Judgement in Borderline Personality Disorder

**DOI:** 10.1371/journal.pone.0073440

**Published:** 2013-11-06

**Authors:** Katie Nicol, Merrick Pope, Reiner Sprengelmeyer, Andrew W. Young, Jeremy Hall

**Affiliations:** 1 Division of Psychiatry, University of Edinburgh, Edinburgh, United Kingdom; 2 Royal Edinburgh Hospital, Edinburgh, United Kingdom; 3 School of Psychology, University of St Andrews, St. Andrews, United Kingdom; 4 Department of Psychology, University of York, York, United Kingdom; 5 Neuroscience and Mental Health Research Institute, Cardiff University, Cardiff, United Kingdom; University Of São Paulo, Brazil

## Abstract

**Background:**

Borderline personality disorder (BPD) is a common and serious mental illness, associated with a high risk of suicide and self harm. Those with a diagnosis of BPD often display difficulties with social interaction and struggle to form and maintain interpersonal relationships. Here we investigated the ability of participants with BPD to make social inferences from faces.

**Method:**

20 participants with BPD and 21 healthy controls were shown a series of faces and asked to judge these according to one of six characteristics (age, distinctiveness, attractiveness, intelligence, approachability, trustworthiness). The number and direction of errors made (compared to population norms) were recorded for analysis.

**Results:**

Participants with a diagnosis of BPD displayed significant impairments in making judgements from faces. In particular, the BPD Group judged faces as less approachable and less trustworthy than controls. Furthermore, within the BPD Group there was a correlation between scores on the Childhood Trauma Questionnaire (CTQ) and bias towards judging faces as unapproachable.

**Conclusion:**

Individuals with a diagnosis of BPD have difficulty making appropriate social judgements about others from their faces. Judging more faces as unapproachable and untrustworthy indicates that this group may have a heightened sensitivity to perceiving potential threat, and this should be considered in clinical management and treatment.

## Introduction

Borderline Personality Disorder (BPD) is a common and serious mental illness that affects about 1-2% of the general population, up to 25% of psychiatric inpatients, and 10% of outpatients [1,2]. The exact causes of BPD are not known, but both genetic and environmental risk factors are believed to contribute to the aetiology of the disorder [1,2], with childhood trauma showing a particularly strong association with the later development of BPD [2,3]. Individuals with a diagnosis of BPD often have difficulties in forming and maintaining interpersonal relationships, and such social impairments are a diagnostic feature of the disorder [4]. Difficulties in social interaction, as seen in BPD, can make maintaining a job or achieving sustained success in the workplace or in education challenging. Furthermore social difficulties experienced by individuals with BPD often show little or no improvement with time [5]. In the current study we sought to characterise social cognition in BPD using well-validated tests of social judgement from faces.

The term 'social cognition' refers to the way in which we gather information about the people around us, allowing us to make judgements and inferences about their characteristics, including personality and intentions [6]. Key to social cognition is the ability to judge how people may feel or think in a given situation [7-10]. Effective social functioning requires the involvement of a network of brain areas in order to gather, retrieve and process relevant information in the correct way, and is fundamental not just in cultivating relationships with others, but for everyday functioning and social interaction of all kinds. A very important source of social information is the face, and consequently, one major means through which social brain function has been investigated in healthy volunteers and patient groups has been through the study of face perception [11]. Investigations of social perception from faces have implicated several brain areas as important in social cognition, including the medial frontal cortex, the amygdala, the insula, the cingulate cortex and precuneus, regions of superior temporal cortex and the fusiform gyrus [7,12-16]. 

Previous studies have investigated social cognition in BPD using a range of measures. Studies of facial expression recognition in BDP to date have recorded inconsistent findings - two studies reported impairments in recognising facial emotions [17,18], while another study found no significant deficit of facial emotion identification, but noted a tendency of individuals with BPD to rate ambiguous faces as negative or aversive [19]. Three studies further identified a more intense response of those with BPD to images of negative facial emotion compared to positive, a finding which was not evident in control participants [17,18,20]. Using interview-based methods Hill and colleagues demonstrated impairments in social function in BPD [21]. Preißler and colleagues also found evidence of social cognition deficits in BPD when asked to attribute mental states to characters in short films [22]. Two studies have investigated social cognition in BPD using the ‘Reading the Mind in the Eyes’ test (RMET), which involves participants inferring the mental state of individuals by viewing pictures of the eye region. Of the studies, one reported no difference between BPD and control participants [22], although this group did question the ecological validity of the test, and the other reported enhanced sensitivity to social characteristics in BPD [23]. The apparent inconsistencies in the literature may be explained by inherent task differences, with conditions in the RMET less representative of a “real life” or familiar situation than in the other tasks described. Fertuck and colleagues also acknowledge that the RMET represents a very low stress task and does not allow for the detection of negativity bias [23]. Findings of altered social cognition in BPD are consistent with structural imaging studies of the disorder, which have found that brain areas involved in social cognition, including frontal and medial temporal lobe regions, differed from that of healthy participants [24]. 

Because previous studies of social cognition in BPD have failed to identify consistent findings, we sought to introduce a more systematic approach involving a variety of social inferences commonly made to faces using a well established task [29-31]. Hence, in order to arrive at a more precise characterisation of areas of relative strength and weakness in social decision making in individuals with BPD, we chose characteristics that included those with a clear and relatively well-established physical basis (age, distinctiveness, attractiveness) and those with a more purely social function involving an inference about a psychological trait or disposition (intelligence, approachability, trustworthiness). Considering previous literature, we hypothesised that differences between groups would be evident in those judgements that required inferences about social characteristics and, in particular, that the BPD group would perform significantly poorer that controls in judgements related to the perception of threat (approachability and trustworthiness). We further investigated the association of social performance with clinical measures and measures of childhood trauma in the BPD group. Measuring childhood trauma was of particular interest due to the strong association between childhood adversity and BPD [2,3]. Furthermore, studies of both bipolar disorder and schizophrenia have reported correlations between childhood experience and common symptoms of these disorders [32-34].

## Methods

### Ethical approval

The study was approved by The Lothian Research Ethics Committee. All participants gave written informed consent, and were able to withdraw from the study at any time. Participants who withdrew from the study remained eligible for all treatments (where required) and were not disadvantaged in any way. Anonymised study data can be made available upon application to the authors.

### Participants and Questionnaires

Twenty patients meeting DSM-IV criteria for borderline personality disorder were recruited for the study from outpatient populations. Diagnosis of BPD was established using the SCID-II interview, and patients were screened for comorbidity using the SCID-I and by review of case notes. Exclusion criteria included a history of bipolar I disorder or schizophrenia, current alcohol or drug dependency, or any neurological illness. Participants were all aged between18 and 65. The BPD group consisted of 15 females and 5 males, mean age 34.3 years (SD 8.5) and a mean IQ of 115.9 (SD 7.4). Of these, 11 were being treated with antipsychotic medication, and 13 were being treated with antidepressant medication. Healthy controls were recruited from community volunteers, did not have a diagnosis of BPD (confirmed on interview). Exclusion criteria were the same as for the BPD participants. The control group consisted of 16 females and 5 males, mean age of 34.5 years (SD 11.6) and mean IQ of 114.2 (SD 7.3). All participants were asked to complete the National Adult Reading Test (NART) as an estimate of IQ. Participants were also asked to complete the Hamilton Rating Scale for Depression (HAM-D), Young Mania Rating Scale (YMRS) and Childhood Trauma Questionnaire (CTQ). The CTQ is a self-report measure of childhood experience, consisting of 18 statements, each relating to one of five subscales of neglect or abuse (eg “I didn't have enough to eat”). Participants rated each question based on the frequency it was experienced throughout childhood and adolescence from a choice of five responses - never true, rarely true, sometimes true, often true, very often true.

### Social judgement

Participants completed a test of social judgement of faces, which consisted of six separate blocks with 32 trials each. In each trial, a different photograph of a face was presented. Participants had to judge whether the faces presented were 'high' or 'low' on a specified characteristic [30,]. In each block, a different characteristic had to be judged, these characteristics were age, distinctiveness, attractiveness, intelligence, approachability and trustworthiness. 

Only a single characteristic was judged in each block. Faces appeared on a computer screen for 3 seconds each and participants were asked to choose from two options for the judged characteristic (eg “trustworthy” or “untrustworthy”). Judgements were reported verbally to the experimenter, and there was no limit on how long participants were given to make a choice, but the next picture was not shown until a judgement had been made. Answers were recorded by the experimenter but no feedback was provided. Participants were given 8 practice trials before each experimental block; practice trials were excluded from analysis.

The stimuli were colour photographs of Caucasian male and female adult faces selected from a previously collected database of 1000 photographs of faces of non-famous people. All the pictures were cropped around the face and hair, so that the minimum possible clothing and background were visible. The photographs were all adjusted to the same height (150 pixels; approximately 5 cm on the screen display), while the width varied slightly. No other attempt was made to standardise the pictures. Instead, the database included photographs that covered a wide range of adult ages, poses, and expressions, so that as many as possible of the naturally occurring cues would be present in the images. All the photographs had been previously rated on several characteristics with 1 to 7 point scales for all characteristics. A mean rating was then computed for each facial stimulus on each characteristic and the selection of the stimuli for the present experiments was based on those mean ratings. A set of 32 faces was selected for each characteristic. Each individual face appeared only in one set; i.e. completely different faces were selected for the sets of faces involving judgments of age, sex, attractiveness, etc. For age, distinctiveness, attractiveness, approachability, intelligence, and trustworthiness, there were 16 faces that had been rated high and 16 faces that had been rated low on the respective characteristics, half of which were male and half female [30].

For analysis, the total number of correct and incorrect judgements for each of the six characteristics was recorded for each participant, as well as the direction of decision bias – for example trustworthy faces being judged as untrustworthy, or vice versa. Note that the sense in which a judgement is considered correct is therefore simply that it is in agreement with the set of ratings from which the faces were chosen (that is, a highly rated face judged as 'high' is considered a correct judgement, and a low rated face judged as 'low' is likewise considered a correct judgement). In this way, the measure used indicates how closely a participant's responses correspond to those of a normal perceiver.

### Statistical Analysis

Statistical analysis was carried out using IBM SPSS, version 19.0 for Windows. T-tests were used to investigate mean differences between the BPD and control groups in age, IQ, YMRS, HAM-D and CTQ scores. Repeated measures ANOVAs were used to investigate performance in the social judgment tasks with judged characteristic as the within subject and group as the between subject factor. To explore group effects in more detail, we used t-tests. In order to correct for multiple comparisons only effects that survived False Discovery Rate correction [31] at p < 0.05 were considered significant. 

## Results

### Demographic Characteristics

There was no significant difference in age (t_1,39_ = -0.07, p = 0.94) or IQ (t_1,37_ = 0.73, p = 0.47) between the control and BPD groups (Table 1). However two individuals from the BPD group and one from the control group chose not to complete the NART, and so were excluded from IQ analysis. The BPD group scored significantly higher than controls on the HAM-D (t_1,39_ = 7.77, p < 0.001), the YMRS (t_1,39_ = 4.83, p < 0.001) and the CTQ (t_1,39_ = 9.39, p < 0.001) (Table 1).

**Table 1:** Demographics.

**Characteristic**	**Participants with diagnosis of BPD (n=20)**	**Healthy control participants (n=21)**
Age	34.3 (SD 8.5)	34.5 (SD 11.6)
Sex	Female=15 Male=5	Female=16 Male=5
IQ (NART)	115.9 (SD 7.4)	114.2 (SD 7.3)
Handedness	Right=17 Left=2 Mixed=1	Right=18 Left=3 Mixed=0
Number of BPD criteria	7.4 (SD 1.3)	0
Hamilton Rating Scale for Depression score	14.5 (SD 8.3)	0.3 (SD 0.7)
Young Mania Rating Scale score	2.5 (SD 2.4)	0
Childhood Trauma Questionnaire score	37.4 (SD 17.6)	1.2 (SD 1.7)
Number on one or more antipsychotic medication	11	0
Number on one or more antidepressant medication	13	0
Comorbid diagnoses, current and past (number)	BPADii (4), OCD (2), PTSD (2), eating disorder (6), alcohol dependency (3), panic disorder (1); paranoid personality disorder (1); avoidant personality disorder (1)	None

Table showing population demographics and mean questionnaire scores for control and BPD groups.

### Social judgement

**Figure 1:**
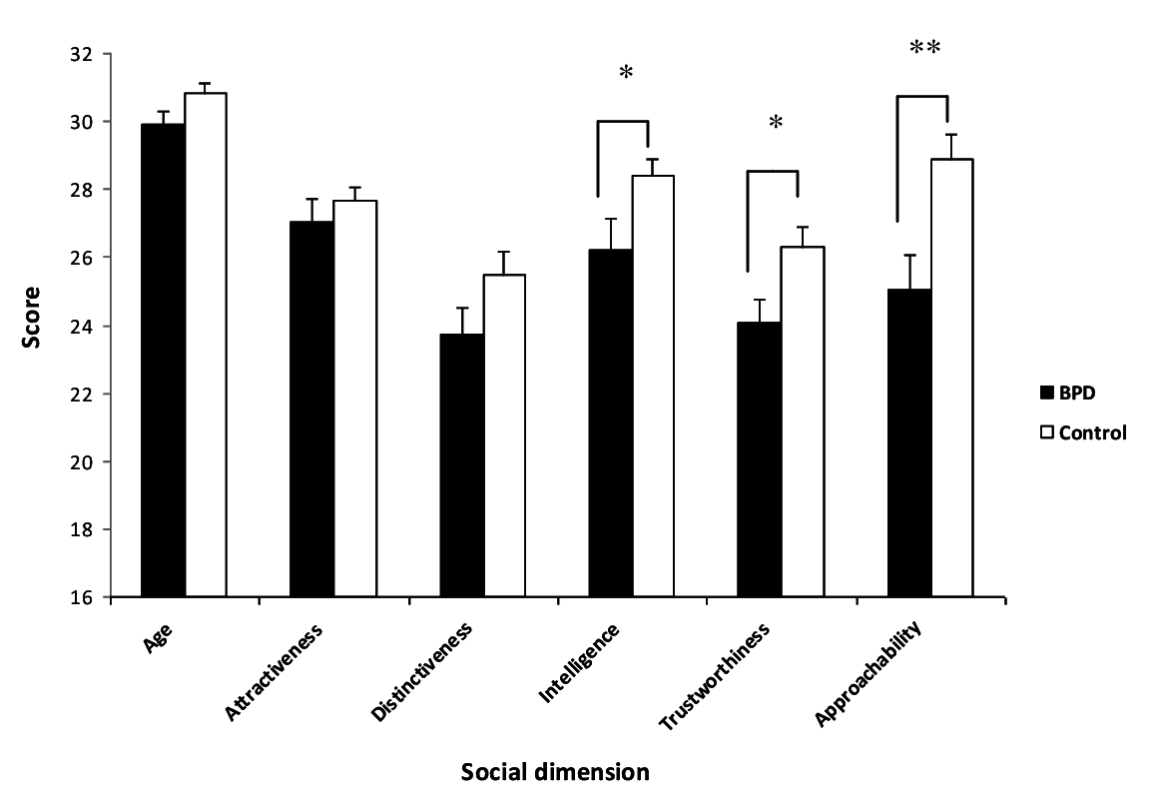
Graph of social judgement scores for each of six dimensions. The BPD group scored significantly lower than the control group on judgements of intelligence, trustworthiness and approachability.

Performance in the test of social cognition was analysed using repeated measures ANOVAs, with group (two levels) as the between subjects factor and judged characteristic (six levels: age; distinctiveness; attractiveness; intelligence; approachability; trustworthiness) as a within subjects factor. There was a significant main effect for group (F_1,39_ = 12.2, p = 0.01) and judged characteristic (F_5,195_ = 23.3, p < 0.01) but no significant interaction between the two (F_5,195_ = 1.9, p = 0.1) (Figure 1). *Post-hoc* t-tests were used to explore which judged characteristics showed significant differences between groups. These revealed that the between-group differences were greatest for the tests of approachability (t_1,39_ = -3.1, p < 0.01), trustworthiness (t_1,39_ = -2.5, p < 0.05) and intelligence (t_1,39_ = -2.1, p < 0.05), but were not significant for the other social dimensions (age (t_1,39_ = -1.8, p = 0.09); attractiveness (t_1,39_ = -0.81, p = 0.42); distinctiveness (t_1,39_ = -1.67, p = 0.10)) (Figure 1). Only the results for the approachability and trustworthiness tests remained significant at p < 0.05 after FDR correction for the multiple comparisons made.

### Judgement bias analysis

**Figure 2:**
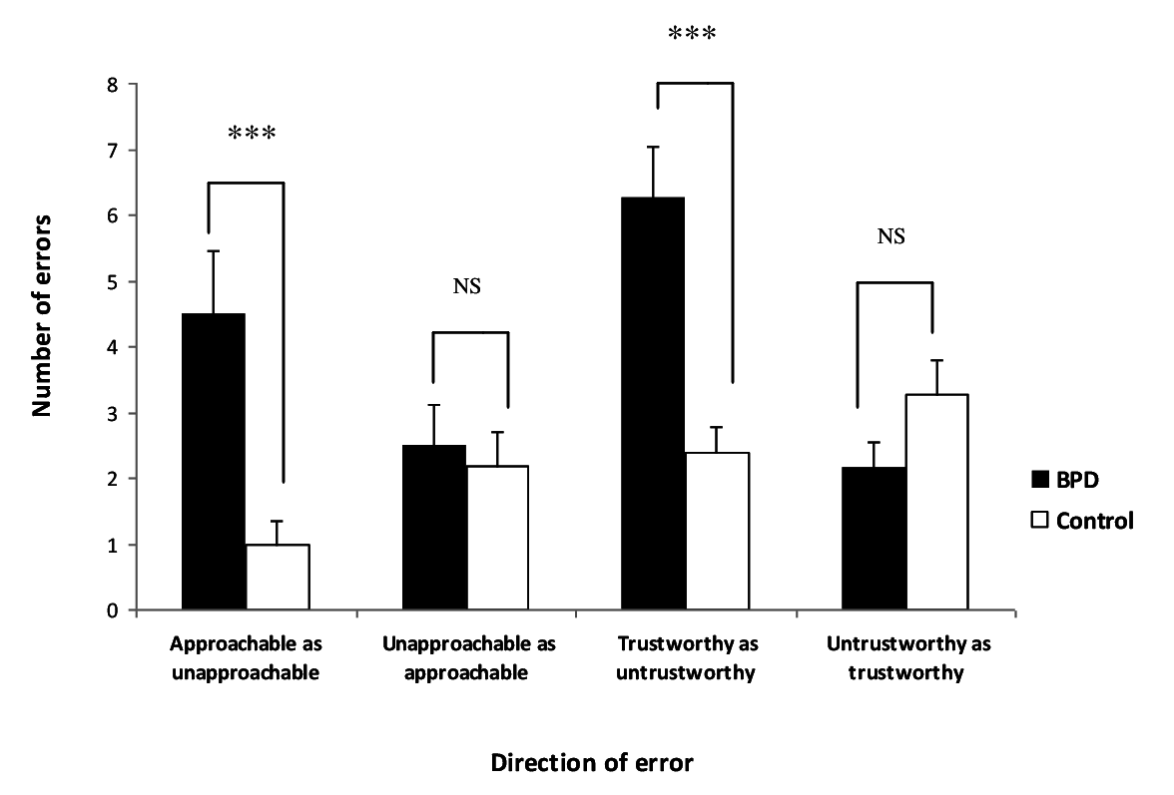
Graph showing the direction of judgement bias for approachability and trustworthiness. The BPD group judged significantly more people as unapproachable and untrustworthy than the control group.

In order to assess whether there was any bias in the direction of social judgements of approachability and trustworthiness made by the BPD group, deviations from the expected answer given by participants when judging approachability and trustworthiness were counted and the direction of change recorded (for example, judging approachable as unapproachable or judging unapproachable as approachable). Repeated measures analysis of variance revealed a group x error direction interaction for judgements of approachability (F_1,39_ = 6.3, p < 0.05) and trustworthiness (F_1,39_ = 14.1, p < 0.01). *Post hoc* t-tests revealed that these effects derived from the BPD group judging more faces as unapproachable (t_1,39_ = 3.5, p < 0.01) and untrustworthy (t_1,39_ = 4.2, p < 0.01) than the control group (Figure 2).

### Correlation analysis

Correlation analysis revealed no significant correlation between the HAM-D, CTQ, or YMRS and the number of errors on each social judgement task made by participants in either the control or BPD group. However, a significant correlation was noted in the BPD group between Childhood Trauma Questionnaire scores and bias towards judging faces as unapproachable (r = .49, p < 0.05). Examination of the subscales of the CTQ revealed that this correlation was strongest for the sexual abuse subscale of the CTQ (r = .53, p < 0.05). No other correlation was found between social judgement bias and clinical measures.

## Discussion

Participants with a diagnosis of BPD performed less well than controls in a test of social judgement from faces. Those in the BPD group scored lower overall than healthy participants and, in particular, this group had difficulty judging trustworthiness and approachability. Notably, the BPD group judged more faces as unapproachable and untrustworthy than the control group. Furthermore, bias towards judging approachable faces as unapproachable in those with BPD correlated with childhood trauma scores as measured by the CTQ.

Both approachability and trustworthiness judgements are related to threat detection, as incorrectly judging a person as approachable or trustworthy could have potentially hazardous consequences. Although efficient social functioning requires a network of brain areas for information processing, it is the amygdala that is principally involved in fear and threat detection, and this area has been shown consistently to be necessary for normal social cognition in animal and human studies [35]. There is evidence from neuropsychological studies [12] that the amygdala plays a role in social judgements and functional brain imaging studies have also reported amygdala involvement in the judgement of trustworthiness [13,] and approachability [31]. Martens and colleagues [37] also noted a relationship between amygdala volume and approachability in patients with Williams syndrome, with increased amygdala volume correlating with higher approachability ratings of faces. Patients with BPD have been shown to have decreased amygdala volume in structural MRI studies, and heightened amygdala responses to facial stimuli in functional MRI studies [26,28,,39,]. Furthermore, previous studies of facial emotion recognition in BPD have suggested that individuals with BPD may show an increased tendency to rate neutral faces as negative or threatening [17,19]. The present findings are therefore consistent with suggestions of heightened amygdala-mediated threat responses to facial and social stimuli in BPD.

Todorov and colleagues [41,], have developed an important perspective on how social characteristics are evaluated in faces that suggests they are based on orthogonal dimensions involving the appraisal of valence (positive or negative intentions) and dominance (ability to enact intentions). In this approach, the appraisal of trustworthiness and approachability are closely linked to the valence factor, raising the interesting possibility for future research that it may be this factor that is particularly affected in BPD.

Our findings also indicate that skewed perception of approachability in those with a diagnosis of BPD is specifically related to childhood adversity, suggesting that trauma in childhood has a sustained and lasting impact on social cognition in BPD. Although the neurobiological substrates of this effect are not known, childhood adversity has recently been related to enhanced amygdala reactivity in a healthy population sample [39] and in major depressive disorder [42], suggesting that childhood trauma may alter social judgement in BPD by modulating amygdala activation [43].

The current results are also consistent with a broader dysfunction in brain networks sub-serving social cognition and mentalising in BPD. Previous studies in healthy individuals have demonstrated the importance of a number of brain regions in social judgement including the medial prefrontal cortex, inferior prefrontal cortex, cingulate cortex and superior temporal cortex [13,16,,]. Structural imaging studies of patients with BPD have also shown altered structure of distributed brain regions implicated in social cognition including reductions in volumes of regions of the frontal and medial temporal lobes [24]. Furthermore, dysfunctional connectivity between the amygdala and PFC has been reported in studies of BPD [45,]. Taken together with these previous findings, our present results suggest negative bias in approachability and trustworthiness judgements may derive from a wider disruption of fronto-limbic circuits in BPD, with a failure of frontal regions to regulate affective responses in individuals with the disorder.

The current findings suggest that individuals with BPD display differences in social cognition which are distinct from those seen in other neuropsychiatric disorders. The pattern of results we observed in the tests of social judgement in individuals with BPD differed from those seen on the same tests in patients with schizophrenia [29], patients with autism [46], and patients with depression (Hall et al, unpublished data). These results suggest a degree of specificity to the breakdown of social inferences in individuals with BPD, which may be particularly related to increased sensitivity to threat. 

In a previous study of social cognition in BPD Fertuck and colleagues found an increased ability of their BPD group to correctly identify the mental state of others using the ‘Reading the Mind in the Eyes’ test [23]. Whilst these results may appear contradictory to the present findings, this is not necessarily the case. Heightened responsiveness of those with BPD to certain types of social cues, such as perceived threat in social situations, could contribute to both findings. Thus in our task (with dichotomous choices on a single characteristic) a heightened sensitivity to certain types of cues would be evident as biased performance (and hence a lower score on a given characteristic). However in the ‘Reading the Mind in the Eyes’ test, where the participant is given the choice of four potential social labels which are not related, heightened sensitivity to one particular dimension could appear as improved overall performance. However in the future, a study administering both tests to the same group would be helpful to further reconcile these results and to understand the possible causes of any apparent discrepancy in their findings.

The heightened responsiveness of patients to potentially threatening situations, illustrated by increased judgements of trustworthy faces as untrustworthy and approachable faces as unapproachable, highlights the social difficulties experienced by those with a diagnosis of BPD, and has clinical implications for the management of patients. Individuals with BPD appear more likely to view neutral situations as threatening, and may require additional reassurance and assistance in understanding social cues. In addition, it appears that childhood trauma is an important risk factor in the development of BPD, and earlier intervention or symptom treatment may be prudent to prevent the disorder, or to minimise the difficulties experienced. It has been suggested that disruption in mentalisation may underpin many of the core features of BPD, including social difficulties [9], and our results are consistent with this idea, supporting the potential value of mentalisation based therapies as a treatment for individuals with a diagnosis of BPD [47].

## References

[1] Leichsenring F, Leibing E, Kruse J, New AS, Leweke F (2011) Borderline personality disorder. Lancet 377: 74-84. doi:10.1016/S0140-6736(10)61422-5. PubMed: 21195251. 21195251

[2] Lieb K, Zanarini MC, Schmahl C, Linehan MM, Bohus M (2004) Borderline personality disorder. Lancet 364: 453-461. doi:10.1016/S0140-6736(04)16770-6. PubMed: 15288745. 15288745

[3] Ball JS, Links PS (2009) Borderline personality disorder and childhood trauma: evidence for a causal relationship. Curr Psychiatry Rep 11(1): 63-68. doi:10.1007/s11920-009-0010-4. PubMed: 19187711. 19187711

[4] American Psychiatric Association (2000) Diagnostic and statistical manual of mental disorders (revised 4th ed.). Washington, DC: Author.

[5] Zanarini MC, Frankenburg FR, Bradford Reich D, Fitzmaurice G (2010) The 10-year course of psychosocial functioning among patients with borderline personality disorder and axis II comparison subjects. Acta Psychiatr Scandanavia 122: 103-109. doi:10.1111/j.1600-0447.2010.01543.x. PubMed: 20199493. PMC3876887 20199493

[6] Billeke P, Aboitiz F (2013) Social cognition in schizophrenia: from social stimuli processing to social engagement. Front Psychiatry 4(4). doi:10.3389/fpsyt.2013.00004. PMC3580762 23444313

[7] Amodio DM, Frith CD. (2006) (2006) Meeting of minds: the medial frontal cortex and social cognition. Nat Rev Neurosci 7: 268-277. doi:10.1038/nrn1884. PubMed: 16552413. 16552413

[8] Choi-Kain LW, Gunderson JG (2008) Mentalisation: Ontogeny, assessment, and application in the treatment of borderline personality disorder. Am J Psychiatry 165: 1127-1135. doi:10.1176/appi.ajp.2008.07081360. PubMed: 18676591. 18676591

[9] Fonagy P, Luyten P, Strathearn L (2011) Borderline personality disorder, mentalisation, and the nuerobiology of attachment. Infant Ment Health J 32(1): 47-69. doi:10.1002/imhj.20283. 28543560

[10] McDonald S (2013) Impairments in social cognition following severe traumatic brain injury. J Int Neuropsychol Soc 19: 1-16. doi:10.1017/S1355617713000428. PubMed: 23351330. 23351330

[11] Young AW, Bruce V (2011) Understanding person perception. Br J Psychol 102: 959-974. doi:10.1111/j.2044-8295.2011.02045.x. PubMed: 21988395. 21988395

[12] Adolphs R, Tranel D, Damasio AR (1998) The human amygdala in social judgment. Nature 393: 470-474. doi:10.1038/30982. PubMed: 9624002. 9624002

[13] Winston JS, O'Doherty J, Dolan RJ (2003) Common and distinct neural responses during direct and incidental processing of multiple facial emotions. NeuroImage 20: 84-97. doi:10.1016/S1053-8119(03)00303-3. PubMed: 14527572. 14527572

[14] Marwick K, Hall J (2008) Social cognition in schizophrenia: a review of face processing. Br Med Bull 88: 43-58. doi:10.1093/bmb/ldn035. PubMed: 18812413. 18812413

[15] Adolphs R (2010) Why does the amygdala contribute to social cognition? Ann N Y Acad Sci 1191: 42-61. doi:10.1111/j.1749-6632.2010.05445.x. PubMed: 20392275. 20392275 PMC2871162

[16] Mar RA (2011) The neural bases of social cognition and story comprehension. Annu Rev Psychol 62: 103-134. doi:10.1146/annurev-psych-120709-145406. PubMed: 21126178. 21126178

[17] Levine D, Marziali E, Hood J (1997) Emotion processing in borderline personality disorders. J Nerv Ment Dis 185(4): 240-246. doi:10.1097/00005053-199704000-00004. PubMed: 9114809. 9114809

[18] Bland AR, Williams CA, Scharer K, Manning S (2004) Emotion processing in borderline personality disorders. Issues Ment Health Nurs 25: 655-672. doi:10.1080/01612840490486692. PubMed: 15371135. 15371135

[19] Domes G, Czieschnek D, Weidler F, Berger C, Fast K et al. (2008) Recognition of facial affect in borderline personality disorder. J Pers Disord 22(2): 135-147. doi:10.1521/pedi.2008.22.2.135. PubMed: 18419234. 18419234

[20] Meyer B, Pilkonis PA, Beevers CG (2004) What’s in a (neutral) face? Personality disorders, attachment styles, and the appraisal of ambiguous social cues. J Pers Disord 18(4): 320-336. doi:10.1521/pedi.18.4.320.40344. PubMed: 15342321. 15342321

[21] Hill J, Pilkonis P, Morse J, Feske U, Reynolds S et al. (2010) Social domain dysfunction and disorganization in borderline personality disorder. Psychol Med 38(1): 135-146. PubMed: 17892627. 10.1017/S0033291707001626 PMC2828321 17892627

[22] Preißler S, Dziobek I, Ritter K, Heekeren HR, Roepke S (2010) Social cognition in borderline personality disorder: evidence for disturbed recognition of the emotions, thoughts, and intentions of others. Front Behav Neuroscience 4: 182. 10.3389/fnbeh.2010.00182 PMC2999836 21151817

[23] Fertuck EA, Jekal A, Song I, Wyman B, Morris MC et al. (2009) Enhanced “Reading the Mind in the Eyes” in borderline personality disorder compared to healthy controls. Psychol Med 39(12): 1979-1988. doi:10.1017/S003329170900600X. PubMed: 19460187. 19460187 PMC3427787

[24] Tebartz van Elst L, Hesslinger B, Thiel T, Geiger E, Haegele K et al. (2003) Frontolimbic brain abnormalities in patients with borderline personality disorder: a volumetric magnetic resonance imaging study. Biol Psychiatry 54(2): 163-171. doi:10.1016/S0006-3223(02)01743-2. PubMed: 12873806. 12873806

[25] Soloff P, Nutche J, Goradia D, Diwadkar V (2008) Structural brain abnormalities in borderline personality disorder: a voxel-based morphometry study. Psychiatry Res 164(3): 223-236. doi:10.1016/j.pscychresns.2008.02.003. PubMed: 19019636. 19019636 PMC3286221

[26] Nunes PM, Wenzel A, Borges KT, Porto CR, Caminha RM et al. (2009) Volumes of the hippocampus and amygdala in borderline personality disorder: a meta-analysis. J Pers Disord 23(4): 333-345. doi:10.1521/pedi.2009.23.4.333. PubMed: 19663654. 19663654

[27] Brunner R, Henze R, Parzer P, Kramer J, Feigl N et al. (2010) Reduced prefrontal and orbitofrontal gray matter in female adolescents with borderline personality disorder: is it disorder specific? NeuroImage 49(1): 114-120. doi:10.1016/j.neuroimage.2009.07.070. PubMed: 19660555. 19660555

[28] Mauchnik J, Schmahl C (2010) The latest neuroimaging findings in borderline personality disorder. Curr Psychiatry Rep 12: 46-55. doi:10.1007/s11920-009-0089-7. PubMed: 20425310. 20425310

[280] Hall J, Olabi B, Lawrie SM, McIntosh AM (2010) Hippocampal and amygdala volumes in borderline personality disorder: a meta-analysis of magnetic resonance imaging studies. Pers Ment Health 4(3): 172-179. doi:10.1002/pmh.128.

[29] Hall J, Harris JM, Sprengelmeyer R, Sprengelmeyer A, Young AW et al. (2004) Social cognition and face processing in schizophrenia. Br J Psychiatry 185: 169-170. doi:10.1192/bjp.185.2.169. PubMed: 15286070. 15286070

[30] Santos IM, Young AW (2008) Effects of inversion and negation on social inferences from faces. Perception 37: 1061-1078. doi:10.1068/p5278. PubMed: 18773729. 18773729

[31] Hall J, Whalley HC, McKirdy JW, Sprengelmeyer R, Santos IM et al. (2010) A common neural system mediating two forms of neural judgement. Psychol Med 40: 1183-1192. doi:10.1017/S0033291709991395. PubMed: 19811702. 19811702

[32] Hammersley P, Dias A, Todd G, Bowen-Jones K, Reilly B et al. (2003) Childhood trauma and hallucinations in bipolar affective disorder: preliminary investigation. Br J Psychiatry 182: 543-547. doi:10.1192/bjp.182.6.543. PubMed: 12777347. 12777347

[33] Read J, van Os J, Morrison AP, Ross CA (2005) Childhood trauma, psychosis and schizophrenia: a literature review with theoretical and clinical implications. Acta Psychiatr Scandanavia 112: 330-350. doi:10.1111/j.1600-0447.2005.00634.x. PubMed: 16223421. 16223421

[34] Varese F, Smeets F, Drukker M, Lieverse R, Lataster T et al. (2012) Childhood adversities increase the risk of psychosis: a meta-analysis of patient-control, prospective- and cross-sectional cohort studies. Schizophr Bull 38(4): 661-671. doi:10.1093/schbul/sbs050. PubMed: 22461484. 22461484 PMC3406538

[35] Adolphs R (2001) The neurobiology of social cognition. Curr Opin Neurobiol 11: 231-239. doi:10.1016/S0959-4388(00)00202-6. PubMed: 11301245. 11301245

[36] Todorov A, Baron SG, Oosterhof NN (2008) Evaluating face trustworthiness: a model based approach. Scan 3: 119-127. PubMed: 19015102. 19015102 10.1093/scan/nsn009 PMC2555464

[37] Martens MA, Wilson SJ, Dudgeon P, Reutens DC (2009) Approachability and the amygdala: insights from Williams syndrome. Neuropsychologia 47: 2446-2453. doi:10.1016/j.neuropsychologia.2009.04.017. PubMed: 19406143. 19406143

[38] Donegan NH, Sanislow CA, Blumberg HP, Fulbright RK, Lacadie C et al. (2003) Amygdala hyperactivity in borderline personality disorder: implications for emotion regulation. Biol Psychiatry 54(11): 1284-1293. doi:10.1016/S0006-3223(03)00636-X. PubMed: 14643096. 14643096

[39] Minzenberg MJ, Fan J, New AS, Tang CY, Siever LJ (2007) Fronto-limbic dysfunction in response to facial emotion in borderline personality disorder: and event-related fMRI study. Psychiatry Res Neuroimging 155(3): 231-243. doi:10.1016/j.pscychresns.2007.03.006. PMC2084368 17601709

[40] Todorov A, Said CP, Engell AD, Oosterhof NN (2008) Understanding evaluation of faces on social dimensions. Trends Cogn Sci 12(12): 455-460. doi:10.1016/j.tics.2008.10.001. PubMed: 18951830. 18951830

[41] Oosterhof NN, Todorov A (2008) The functional basis of face evaluation. Proc Natl Acad Sci USA 105(32): 11087-11092. PubMed: 18685089. 18685089 10.1073/pnas.0805664105 PMC2516255

[42] Dannlowski U, Stuhrmann A, Beutelmann V, Zwanzger P, Lenzen T et al. (2012) Limbic scars: long-term consequences of childhood maltreatment revealed by functional and structural magnetic resonance imaging. Biol Psychiatry 71(4): 286-293. doi:10.1016/j.biopsych.2011.10.021. PubMed: 22112927. 22112927

[43] Grant MM, Cannistraci C, Hollon SD, Gore J, Shelton R (2011) Childhood trauma history differentiates amygdala response to sad faces within MDD. J Psychiatr Res 45(7): 886-895. PubMed: 21276593. 21276593 10.1016/j.jpsychires.2010.12.004 PMC3090525

[44] New AS, Hazlett EA, Buchsbaum MS, Goodman M, Mitelman SA et al. (2007) Amygdala-prefrontal disconnection in borderline personality disorder. Neuropsychopharmacology 32: 1629-1640. doi:10.1038/sj.npp.1301283. PubMed: 17203018. 17203018

[45] Wolf RC, Sambataro F, Vasic N, Schmid M, Thomann PA et al. (2011) Aberrant connectivity of resting-state networks in borderline personality disorder. J Psychiatry Neurosci 36(6): 402-411. doi:10.1503/jpn.100150. PubMed: 21406160. 21406160 PMC3201994

[46] Philip RC, Whalley HC, Stanfield AC, Sprengelmeyer R, Santos IM et al. (2010) Deficits in facial, body movement and vocal emotion processing in autism spectrum disorders. Psychol Med 40(11): 1919-1929. PubMed: 20102666. 20102666 10.1017/S0033291709992364

[47] Bateman A, Fonagy P (2010) Mentalisation based treatment for borderline personality disorder. World Psychiatry 9: 11-15. PubMed: 20148147. 20148147 10.1002/j.2051-5545.2010.tb00255.x PMC2816926

